# Imaging of Atypical and Complicated Posterior Reversible Encephalopathy Syndrome

**DOI:** 10.3389/fneur.2019.00964

**Published:** 2019-09-04

**Authors:** Amin F. Saad, Ruchir Chaudhari, Max Wintermark

**Affiliations:** ^1^Department of Radiology, Baylor University Medical Center, Dallas, TX, United States; ^2^Department of Radiology, Stanford University, Stanford, CA, United States

**Keywords:** PRES (posterior reversible encephalopathy syndrome), encephalopathy, hypertension, intracranial hemorrhage, pathophsiology

## Abstract

Posterior reversible encephalopathy syndrome (PRES) is a condition clinically characterized by headache, altered mental status, seizures, and visual loss and may be associated with systemic hypertension, preeclampsia/eclampsia, chemotherapy, immunosuppressive therapies in the setting of organ transplantation, and uremic encephalopathy. While brain imaging in patients with PRES typically reveals symmetric vasogenic edema within the parietal and occipital lobes, PRES may present with atypical imaging findings such as central brainstem and deep gray involvement without subcortical edema, and even spinal cord involvement. Additionally, PRES may be complicated in some cases by the presence of cytotoxic edema and hemorrhage. This review will serve to summarize the pathophysiologic theories and controversies underlying PRES, imaging features encountered in atypical and complicated PRES, and the implications these findings may have on patient prognosis.

## Introduction

Posterior reversible encephalopathy syndrome (PRES) is a syndrome affecting the CNS with a range of clinical presentations, most often including headache, altered mental status, seizures, and visual loss. PRES was first described in 1996 by Hinchey et al. (1). A multitude of conditions may lead to the development of PRES, with most common etiologies reported including moderate to severe hypertension, preeclampsia/eclampsia, infection with sepsis and shock, autoimmune disease such as systemic lupus erythematosus, multidrug chemotherapy regimens most often in the setting of hematopoietic malignancies, and in the setting of bone marrow and stem cell transplantation (2). The typical CT and MRI imaging features encountered in the setting of PRES consist of near symmetric hemispheric vasogenic edema affecting subcortical white matter and often extending to involve overlying cortex, best demonstrated with FLAIR sequences (3). Diffusion weighted imaging (DWI) usually confirms the vasogenic nature of this edema with absence of restricted diffusion. While variations exist in the most commonly encountered patterns of edema distribution, Bartynski et al. in an analysis of a large cohort of patients, described lesion distribution patterns to include a holohemispheric watershed pattern (22.8% of 136 patients), superior frontal sulcus pattern (27.2%), and a dominant parietal-occipital pattern (22.1%), with partial or asymmetric expression of these primary patterns in 27.9% of patients. Notably, 98% of patients exhibited some degree of involvement of the parietal-occipital regions (4).

## Pathophysiology of PRES

The precise pathophysiologic mechanism underlying the development of PRES remains unknown, and controversy exists regarding competing mechanistic theories. The first theory describes severe hypertension which exceeds the natural autoregulatory limits of the brain (150–160 mm Hg), with resultant injury to the capillary bed, fluid egress, and resultant vasogenic edema. This theory is supported by the common occurrence of hypertension encountered in patients with PRES (50–70%) (5), animal studies demonstrating the development of vasogenic edema and hyperperfusion with experimentally elevated blood pressure (6), and reports of hyperperfusion in patients imaged with Tc99m-HMPAO single-photon emission CT (SPECT) (7). Problems with this theory include the development of PRES in patients with normal or only mildly increased blood pressure, studies demonstrating hypoperfusion in PRES, and a lack of correlation with the degree of brain edema and the severity of hypertension (5).

A competing theory of PRES pathophysiology describes the development of vasoconstriction due to autoregulatory compensation of severe hypertension leading to reduced brain perfusion, ischemia, and the development of vasogenic edema (8). In this theory, if left untreated or severe, the resultant ischemia may go on to frank infarction, with development of diffusion restriction. This theory is supported by the development of PRES in systemic conditions characterized by endothelial injury and a typical lack of severe hypertension such as sepsis, following bone marrow transplantation, and systemic chemotherapy. Additionally, evidence of vasculopathy in the setting of PRES as demonstrated using catheter angiography with vasoconstriction and reduced perfusion supports this theory, as does the common occurrence of PRES imaging features in a watershed distribution. Finally, imaging studies using MR perfusion have demonstrated hypoperfusion in PRES (9, 10).

A third theory attempting to explain the development of PRES is immune system activation with a resultant cascade which induces endothelial dysfunction. In this theory, cytokines such as tumor necrosis factor alpha and interleukin-1 are released due to a systemic insult, which serve to induce expression of adhesion molecules which interact with circulating leukocytes and trigger the release of reactive oxygen species and proteases, leading to endothelial damage and fluid leakage (11). Additionally, these cytokines cause astrocytes to produce vascular endothelial growth factor (VEGF), which causes an increase in blood brain barrier permeability through the weakening of endothelial cell tight junctions, and has been shown to also activate the vesiculo-vacuolar organelle providing a major route for the extravasation of fluids and macromolecules (12). Marra et al. (11) note that increased circulating levels of VEGF in pre-eclamptic patients, a syndrome significantly associated with PRES, result in a 5-fold increase in vascular permeability (13). Increased levels of leukocyte adhesion molecules have also been associated with preeclampsia, allogenic bone marrow transplantation, solid organ transplantation, and infection/sepsis/shock (5). Brain biopsy in a case of PRES following cardiac transplantation showed endothelial activation, T-cell trafficking, and endothelial VEGF expression (14). In this theory, hypertension and vasoconstriction are both consequences and not primary causative factors in PRES pathogenesis (11, 15).

A recently published theory of the pathophysiology of PRES is that of arginine vasopressin (AVP) hypersecretion (16). Multiple clinical conditions associated with the development of PRES, such as eclampsia and sepsis, are associated with AVP hypersecretion. Largeau et al. thus theorize that this increase in AVP secretion or AVP receptor density results in activation of vasopressin V1a with associated cerebral vasoconstriction, endothelial dysfunction, and cerebral ischemia with resultant cytotoxic edema. This may then lead to increased endothelial permeability and subsequent vasogenic edema (16). This theory may open the possibility for pharmacologic therapies for PRES targeting the AVP axis.

## Atypical Regional Involvement in PRES

While PRES most commonly manifests on imaging as subcortical/cortical edema within the cerebral hemispheres with a parietal-occipital predominance and some variable involvement of deep structures as well as the posterior fossa, it may occur in an atypical fashion (**Figure 3**) with isolated involvement of deep gray nuclei, brainstem/cerebellar hemispheres, and exceptionally the spinal cord without cerebral hemispheric involvement. These findings may lead to a diagnostic dilemma, with a delay in diagnosis and reversal of the offending condition potentially leading to a poor patient outcome. In a series of 124 patients with PRES, McKinney et al. noted 4% of patients had imaging findings of a “central variant” PRES, revealing brainstem or deep gray nuclei involvement without involvement of the cerebral hemispheres (17). In an additional series by McKinney et al. (18) consisting of 76 patients, involvement included the thalamus (30.3%), cerebellum (34.2%), brainstem (18.4%), and basal ganglia (11.8%) with unilateral involvement seen in 2.6%. Liman et al. (19) studied a cohort of 96 patients with PRES and found deep gray nuclei involvement in ~25% of patients and infratentorial involvement (predominantly cerebellar and pontine) in more than 50% of patients. These authors found a parieto-occipital pattern in 53%, superior frontal sulcus pattern in 17%, holohemispheric watershed pattern in 17%, and a central pattern in 14%. Another cohort of 50 patients studied by Kastrup et al. (20) demonstrated basal ganglia involvement in 1.6% of patients and cerebellar involvement in 6.5%. In the few reported cases of spinal cord involvement by PRES, all patients demonstrated confluent expansile central cord T2 signal elevation spanning at least four segments, with involvement of the cervicomedullary junction (21). Five of these nine patients had supratentorial involvement, while all revealed brainstem involvement.

## Hemorrhage in PRES

**Figure 1:**
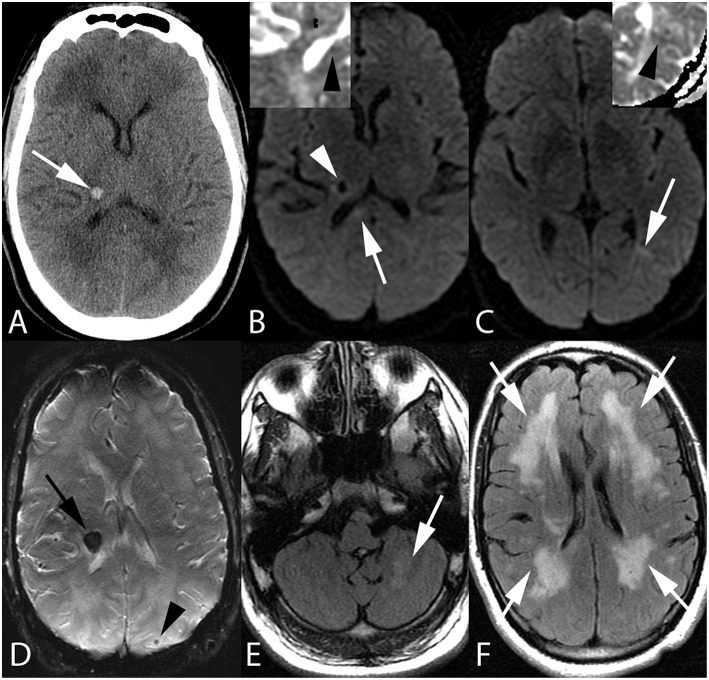
Fifty-five-year-old man with end stage renal disease and severe hypertension. Axial CT image **(A)** reveals a focal parenchymal hemorrhage at the junction of the right thalamus and posterior limb of the right internal capsule (arrow). Axial DWI images with ADC inserts **(B,C)** show foci of diffusion restriction within the right corpus callosum splenium (arrow, **B**) and left temporo-occipital periventricular white matter (arrow, **C**). ADC maps confirm diffusion restriction (insert **B,C**, arrowheads). Again seen is right thalamocapsular hematoma (arrowhead, **B**). Axial SWI image **(D)** demonstrates blooming of right thalamocapsular hematoma (arrow) in addition to a punctate hemorrhage within left parietal subcortical white matter (arrowhead). Axial FLAIR images **(E,F)** show left cerebellar (arrow, **E**) and confluent bilateral frontoparietal (arrows, **F**) edema.

**Figure 2:**
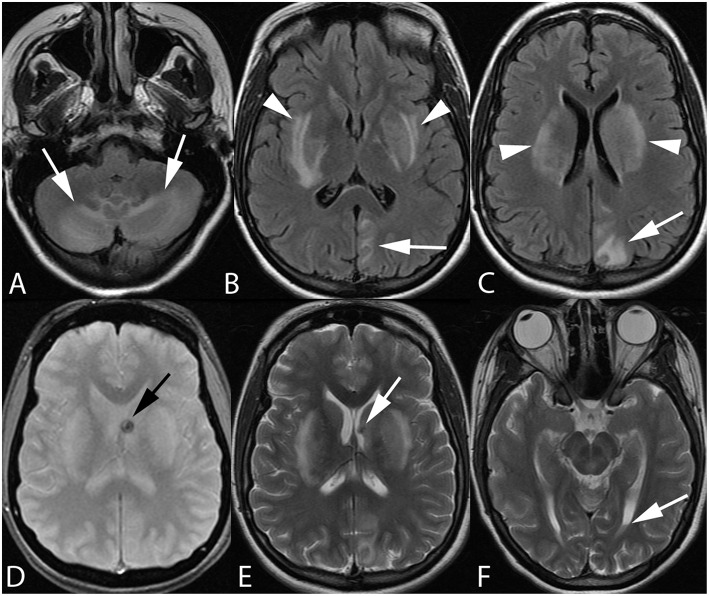
Twenty-one-year-old pregnant woman with eclampsia. Axial FLAIR images **(A–C)** demonstrate bilateral cerebellar hemisphere and vermian (arrows, **A**), bilateral lentiform/caudate and capsular (arrowheads, **B,C**), and left parieto-occipital edema. Axial GRE **(D)** and T2-weighted **(E,F)** images reveal focal hemorrhage within the left caudothalamic groove (arrow, **D,E**) extending to the left lateral ventricular body with a small hematocrit level within the left occipital horn (arrow, **F**) from intraventricular extension of hemorrhage.

**Figure 3:**
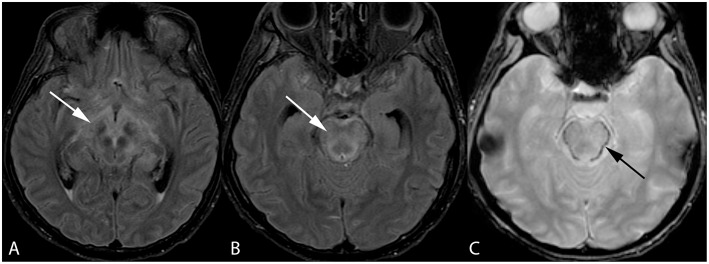
Forty-two-year-old woman with history of bone marrow transplantation. Axial FLAIR images **(A,B)** demonstrate central variant PRES with edema involving the midbrain with extension to the hypothalamus and optic tracts (arrow, **A**) and pons (arrow, **B**). Axial GRE image **(C)** shows petechial hemorrhage at the periphery of the pons (arrow).

Posterior reversible encephalopathy syndrome (PRES) may be complicated by the presence of hemorrhage (Figures 1–3), on the order of 15% in a series of 151 patients studies by Hefzy et al. (22) which utilized gradient echo T2^*^ (GRE) images. In this series, focal petechial/microhemorrhages (<5 mm), sulcal subarachnoid hemorrhage, and focal hematoma formation were seen with equal frequency. Of note, hemorrhage was significantly more common in patients following bone marrow transplantation than in solid organ transplantation, potentially based on underlying coagulopathy, with similar increased incidence in those patients receiving systemic anticoagulation. No difference in hemorrhage incidence was seen in patients with normal, mildly elevated, or severely elevated blood pressure. In a series of 31 patients reported by McKinney et al. (23) utilizing susceptibility-weighted images (SWI), hemorrhage was more commonly detected (64.5% of patients). Microhemorrhages were seen in 58.1% of patients at presentation and 64.7% at follow-up, while subarachnoid hemorrhage was seen in 12.9% and parenchymal hematoma formation was seen in 6.5%. In the series reported by Liman et al. (19), microhemorrhages were seen in 14% of patients, sulcal subarachnoid hemorrhage in 4%, and parenchymal hematoma formation in 11%. Kastrup et al. (20) found microhemorrhages in 17% of the 29 patients who had T2^*^ or SWI images available in their cohort. The overall rate of hemorrhage encountered in PRES range from 15 to 65%, with the majority likely reflecting the majority of the higher reported incidences (24). The mechanism of hemorrhage in PRES may be secondary to pial vessel rupture in the setting of severe hypertension or reperfusion injury in the setting of vasoconstriction (25).

## Diffusion Restriction in PRES

Vasogenic edema predominates in PRES, however cases may be complicated by the development of cytotoxic edema as indicated by diffusion restriction (Figure 1). Some cases may show reversibility of diffusion restriction similar to findings seen in patients with transient cerebral ischemia, venous ischemia/infarction, and vasospasm following subarachnoid hemorrhage although restriction often progresses to frank infarction with encephalomalacia identified on follow-up. In a series of 76 patients reported by McKinney et al. (18), 17.3% demonstrated areas of restricted diffusion. Covarrubias et al. (26) reported a series of 22 patients with PRES, with six patients (22%) demonstrating abnormal diffusion signal and two patients revealing progression to infarction on follow-up. In the setting of extensive vasogenic edema encountered in PRES, some areas of cytotoxic edema amidst regions of vasogenic edema may demonstrate isointense ADC signal, representing ADC pseudonormalization (24).

## Contrast Enhancement in PRES

Contrast enhancement has been variably reported in the setting of PRES, typically presenting as leptomeningeal or gyral cortical enhancement (24). Enhancement was seen in 37.7% of patients studied by McKinney et al. (18), who also reported the rare occurrence of deep white matter or overlying dural enhancement. Karia et al. (27) reported enhancement in 43.7% of 135 patients studied, with a leptomeningeal pattern in 17.8% and a leptomeningeal plus cortical pattern in 15.6%. These authors found no significant association between the presence or pattern of enhancement and patient outcome of MR imaging severity of PRES.

## Vasospasm in PRES

Vasculopathic changes are commonly encountered on vessel imaging in PRES patients. Bartynski et al. (28) found evidence of diffuse vasoconstriction, focal vasculopathy, or vessel pruning in 87% of 46 patients studied with catheter and/or MR angiography (MRA). Of 11 patients with follow up MRA examinations, seven patients revealed improvement or resolution of vasculopathic changes. It is important to note the similarity of these findings with those encountered in reversible cerebral vasoconstriction syndrome (RCVS), which shares significant clinical and radiologic features with PRES (24). Additionally, 9–38% of patients with RCVS demonstrate reversible vasogenic edema (29, 30). The underlying etiologic theories of RCVS include disturbance of cerebrovascular tone and endothelial dysfunction, similar to theories of PRES pathogenesis, and the two diagnoses may reside along a spectrum of manifestations of abnormal cerebral autoregulation and/or endothelial damage (31).

## Establishing Patient Prognosis in PRES

Although PRES is typically reversible (70–90% of cases) (24) and patient prognosis is often positive with removal of the offending condition leading to PRES, complication by hemorrhage and/or diffusion restriction often portends a poorer patient prognosis. In the series reported by Hefzy et al. (22), 23% of patients with PRES complicated by hemorrhage had a poor clinical outcome, with death of six of the seven patients. In the series by Covarrubias et al. (26), death was seen in 50% of the patients who exhibited diffusion signal changes. Additionally, brainstem involvement by PRES is associated with a poorer outcome, with two of three patients who died despite having no diffusion changes in the series by Covarrubias et al. (26) demonstrating extensive brainstem edema. In a review of PRES cases performed by Schweitzer et al. (32), 99 cases of PRES were analyzed for vasogenic edema, hemorrhage, and diffusion restriction. Areas of vasogenic edema were given discrete variables from 1 to 10 based on regional involvement, and the term “extensive vasogenic edema” was defined as involvement of five or more areas. Hemorrhage was categorized based on the presence or absence of mass effect, and diffusion restriction was confirmed with ADC maps. “Advanced radiologic PRES” was defined as at least one of the following: extensive vasogenic edema, diffusion restriction, or hemorrhage with mass effect. Patient outcomes were based on discharge disposition: home or rehabilitation vs. death or hospice, as well as modified Rankin scale (mRS) with an mRS of 3–6 considered a poor outcome. These investigators found that extensive vasogenic edema, presence of hemorrhage, and diffusion restriction (all criteria for “advanced radiologic PRES”) were associated with poor clinical outcomes in terms of both hospital discharge and mRS. In this study, brainstem edema was not associated with a poor mRS at discharge.

## Conclusion

Posterior reversible encephalopathy syndrome (PRES) is a condition commonly encountered in clinical practice, with prompt recognition and intervention to remove precipitating factors serving to optimize patient outcomes and reverse symptoms as well as imaging changes. The recognition of atypical imaging manifestation of PRES is important to avoid delays in diagnosis and treatment, as is identification of complicating factors which may adversely affect patient prognosis.

## Author Contributions

AS wrote the majority of the manuscript. RC wrote portions of the manuscript and supplied case material. MW provided guidance and feedback in the manuscript preparation and research on the topic.

### Conflict of Interest Statement

The authors declare that the research was conducted in the absence of any commercial or financial relationships that could be construed as a potential conflict of interest. The handling Editor declared a past collaboration with one of the authors MW.
